# Role of Cytochrome P450 Enzymes in Plant Stress Response

**DOI:** 10.3390/antiox9050454

**Published:** 2020-05-25

**Authors:** Balaji Aravindhan Pandian, Rajendran Sathishraj, Maduraimuthu Djanaguiraman, P.V. Vara Prasad, Mithila Jugulam

**Affiliations:** 1 Department of Agronomy, Kansas State University, Manhattan, KS 66506, USA; aravindhan@ksu.edu (B.A.P.); rajendransathishraj@ksu.edu (R.S.); jani@tnau.ac.in (M.D.); vara@ksu.edu (P.V.V.P.); 2 Department of Crop Physiology, Tamil Nadu Agricultural University, Coimbatore, Tamil Nadu 641003, India

**Keywords:** cytochrome P450, plant metabolism, antioxidants, plant stress response

## Abstract

Cytochrome P450s (CYPs) are the largest enzyme family involved in NADPH- and/or O_2_-dependent hydroxylation reactions across all the domains of life. In plants and animals, CYPs play a central role in the detoxification of xenobiotics. In addition to this function, CYPs act as versatile catalysts and play a crucial role in the biosynthesis of secondary metabolites, antioxidants, and phytohormones in higher plants. The molecular and biochemical processes catalyzed by CYPs have been well characterized, however, the relationship between the biochemical process catalyzed by CYPs and its effect on several plant functions was not well established. The advent of next-generation sequencing opened new avenues to unravel the involvement of CYPs in several plant functions such as plant stress response. The expression of several CYP genes are regulated in response to environmental stresses, and they also play a prominent role in the crosstalk between abiotic and biotic stress responses. CYPs have an enormous potential to be used as a candidate for engineering crop species resilient to biotic and abiotic stresses. The objective of this review is to summarize the latest research on the role of CYPs in plant stress response.

## 1. Introduction

**Figure 1:**
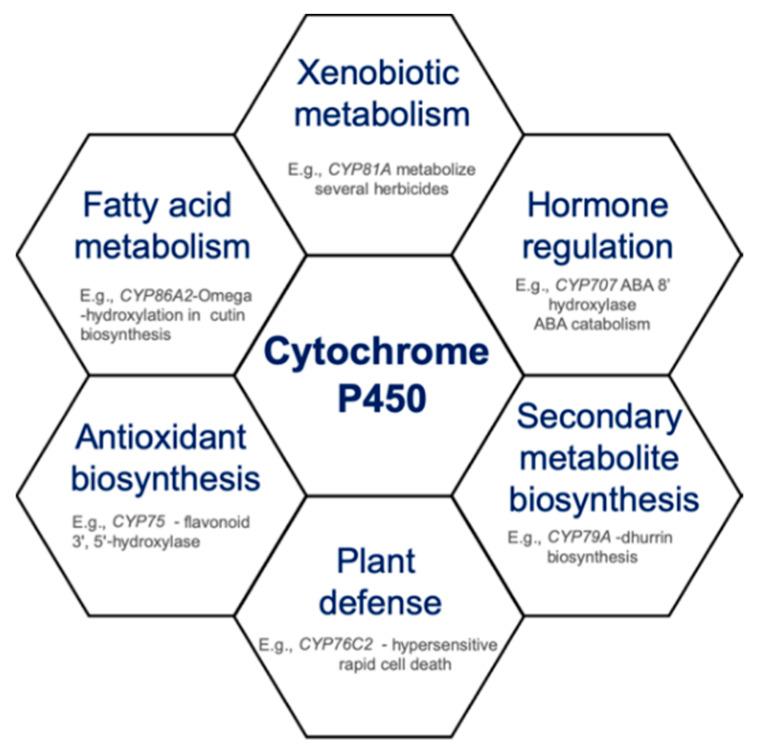
Diverse roles of cytochrome P450s in plants.

Cytochrome P450s (hereafter referred to as CYPs) belong to the oxidoreductases class of enzyme, which represents one of the largest enzyme families containing heme-thiolate as a cofactor. CYPs catalyze NADPH- and/or O_2_-dependent hydroxylation reactions in primary and secondary metabolism in many organisms. CYPs are considered as one of the main contributors to the diversity of metabolites formed through oxidation, reduction, hydroxylation, epoxidation, dealkylation, C–C cleavage, desaturation, decarboxylation, dimerization, isomerization, and ring extension reactions [1]. CYPs are mainly anchored to the endoplasmic reticulum to play an essential role in the biosynthesis of different metabolites [2]. The involvement of CYPs in xenobiotic metabolism is well characterized across microorganisms, insects, plants, and humans, imparting resistance to antibiotics, insecticide, herbicide, and drugs, respectively. Further, the CYPs play diverse roles in plants beyond xenobiotic metabolism, including the biosynthesis of hormones, fatty acids, sterols, cell wall components, biopolymers, and several defense compounds (terpenoids, alkaloids, flavonoids, furanocoumarins, glucosinolates, allelochemicals) (Figure 1). CYPs are also implicated in protecting plants from harsh environmental conditions [3], by enhancing the activity of compounds (e.g., flavonoids) with an increased antioxidant activity [4].

The identification and characterization of CYPs can be divided into pre- and post-genomic eras. In the pre-genomic era, the involvement of CYPs was demonstrated by biochemical techniques such as the isolation of CYPs from microsomal fractions, and the inhibition of CYP activity. Being a large gene family with diverse isoforms, the characterization of CYPs with these methods can be challenging because, in most cases, the substrate of the enzyme cannot be easily predicted [5]. However, in the pre-genomics era, the biochemical and molecular processes of CYPs were characterized; however, the relationship between the biochemical process and its direct and indirect effects on several plant functions was not established. Later, with the advent of next-generation sequencing technology (NGS) followed by its rapid advancement and affordability, new avenues to unravel the involvement of CYPs in several plant functions including stress responses were created. Abiotic/biotic stress can be defined as any non-living/living factor(s) that negatively impact the growth and development of plants. The major abiotic stresses affecting, specifically the crop plants include their response to drought, salinity, high/low temperature, heavy metal toxicity, and herbicide application. On the other hand, the major biotic stresses that affect plants include the infestation of insects, pathogens, or weeds. As plants are sessile, they are forced to respond to dynamic environmental changes to sustain their growth and development. Plants can function normally under optimal environmental conditions; however, they are often exposed to a variety of abiotic/biotic stresses or combinations of both [6]. Such exposure can overwhelm their natural defense systems and may result in a substantial yield loss in crops [7]. CYPs have been found to play a major role in hormone signaling, thereby regulating plant response under stress conditions [8,9,10]. CYPs have been reported to protect plants from drought [8], heat [11], salt [12], heavy metal stress [13], and insects [14] and diseases [15] infestations. In addition, CYPs are also directly involved in the secondary metabolism of plants, facilitating detoxification of external compounds or those that are produced as a byproduct of metabolism in response to stress [16].

The reference genomes and sequence information of many species has been made available as a result of the advancement of sequencing technology and computational capacity in the last decade. Over 300,000 CYP gene sequences have been identified in different organisms, which include ~16,000 plant CYPs [17]. NGS-based technologies such as RNA-Seq, exome seq, and genotyping by sequencing (GBS) combined with several fine mapping methods helped to deduce the function of CYPs. Specific CYP genes involved in plant stress response have also been identified. Such CYP genes have a great potential to be used as candidates for engineering crop species resilient to biotic and abiotic stress. The objective of this review is to summarize the research on the involvement of CYPs in the plant stress response.

## 2. Classification & Catalysis of CYPs

### 2.1. Nomenclature and Basic Classification

The structural classification of plant CYPs are based on the similarity of amino acid sequences. The rules for nomenclature and systematic classification of CYPs were set by the “CYP Nomenclature Committee”, which assigns names to new CYP genes and also updates the CYP database [18]. CYPs are hierarchically divided into clans, families, and subfamilies. All the cytochrome enzymes will have a code “CYP” followed by a family number, followed by an alphabet that denotes the subfamily of the enzyme. Those enzymes with a 40% amino acid sequence similarity are considered as members of the same family, and those with a >55% identity are grouped into the same sub-family, and those with a >97% similarity are considered as an allelic variant of the same gene [19]. The primary amino acid sequence similarity between different CYPs may be very low, but their secondary structure is relatively conserved [20]. CYPs can be broadly grouped into four types based on origin viz., animal, fungal, microbial, and plant CYPs.

CYPs from humans, vertebrates, and insects are classified as animal CYPs and have 196 families grouped into 11 clans. Fungal CYPs constitute the largest group among other CYPs, with 276 families grouped into 115 clans. Microbial CYPs are not yet classified completely [21]. The plant CYPs have 47 families grouped into 11 clans. In plants, the CYP genes cover ~1% of their genome, implying the abundance and importance for CYPs in plant function [22]. Clan membership parameters have not yet been clearly defined [23,24]. CYPs have also been classified based on their function using the enzyme commission number (EC). The classification of CYPs by the EC number is determined by the type of reaction they catalyze and the type of electron donor with which they interact [25]. The usual electron donor for microsomal enzymes is NADPH-hemoprotein reductase (EC 1.6.2.4). The reactions involving monooxygenation and formation of a single molecule of water were classified under the class oxidoreductases in the sub-subclass of EC 1.14.14 and EC 1.14.15. These sub-subclasses of enzymes act on paired donors, with an incorporation or reduction of molecular oxygen. The differences within these sub-subclasses of enzymes have been primarily because of the involvement of donor proteins. For example, EC 1.14.14 uses reduced flavin or flavoprotein as a donor (e.g., EC 1.14.14.16, steroid 21-monooxygenase, or CYP45021A2), whereas EC 1.14.15 uses a reduced iron-sulfur protein as a donor (e.g., EC 1.14.15.8, steroid 15*β*-monooxygenase, or CYP106A2). The mitochondrial CYPs utilize a specialized ferredoxin, known as adrenodoxin—an iron-sulfur protein—as their electron donor, and are thus classified under EC 1.14.15 (e.g., EC 1.14.15.15, cholestanetriol 26-monooxygenase, or CYP27A/CYP27A1/CYP27A1’). Those oxidoreductase enzymes involved in the oxidation of a pair of donors, resulting in the reduction of molecular oxygen to two molecules of water, are classified under EC 1.14.19 (e.g., EC 1.14.19.52, camalexin synthase, or CYP71B15). The exceptions to these classifications are CYPs from the CYP74 family that catalyze dehydration reactions that do not require oxygen or an electron donor and are classified under EC 4.2.1 (e.g., EC 4.2.1.121, colneleate synthase, or CYP74D/CYP74D1/CYP74). CYPs that catalyze isomerization reactions have been classified under other intramolecular oxidoreductases (EC 5.3.99). For example, prostaglandin-I synthase (EC 5.3.99.4), or CYP8A1, is an enzyme involved in prostanoid biosynthesis that belongs to the CYP isomerase [25].

### 2.2. Catalysis of CYP Enzymes

The mechanisms of action of CYPs are extensively studied and documented. CYPs were first reported in rats as a carbon monoxide binding pigment, which absorbs light at 450 nm and later named P-450, where P denotes pigment [26]. The CYP enzymes have heme-thiolate as a cofactor centering porphyrin ring, which makes it one of the metalloenzymes involved in the reduction of molecular oxygen. The most conserved region of CYPs is the heme cofactor, which is also the site of catalysis. The conserved regions and the amino acid sequences of many CYPs have been well-characterized [27,28,29,30]. However, the reactions involved in the substrate binding of CYPs are complex and not completely understood [31].

**Figure 2:**
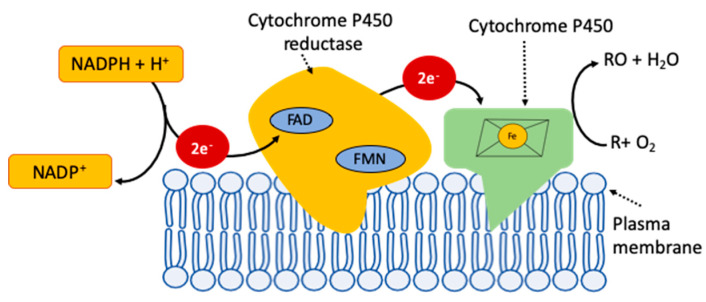
Simplified scheme of catalysis of cytochrome P450 (CYP) system; the CYPs receive two electrons derived from nicotinamide adenine dinucleotide phosphate (NADPH) through cytochrome P450 reductase (CPR) to catalyze the oxidation reaction R + O_2_ + NADPH → RO + H_2_O + NADP^+^; where R is the substrate and RO is the product of the oxidation reaction.

The catalysis of CYPs involves five major steps, step 1: The organic substrate (R) will bind to the heme group of the enzyme; step 2: the substrate binding induces the transfer of an electron from NADPH through cytochrome P450 reductase (CPR) or any other associated reductase to the CYPs that will reduce the iron (Fe) from the ferric state (Fe^3+^) to the ferrous state (Fe^2+^); step 3: molecular oxygen binds to ferrous CYPs to form a ferrous CYP–dioxygen complex; step 4: a second electron is transferred from CPR or any other associated reductase to the ferrous CYP–dioxygen complex to form a short-lived peroxo complex and this complex rapidly protonated twice forming one molecule of water and an iron–oxo complex; step 5: the oxygen atom in the iron–oxo complex binds to the organic substrate (R) and forms the oxidized reaction product (RO) (Figure 2) [32].

R + O_2_ + NADPH → RO + H_2_O + NADP^+^

Apart from oxidation, CYPs are also known to be involved in reactions such as dehydrogenation, carbon–carbon bond cleavage, and dealkylation [33,34,35].

## 3. Role of CYPs in Abiotic and Biotic Stress

### 3.1. Drought Stress

In response to drought (water-deficit) stress, plants trigger multiple enzymatic and hormonal activities to maintain the intracellular ion homeostasis, osmolyte accumulation, and scavenging of ROS such as singlet oxygen (^1^O_2_), superoxide (O_2_^−^), hydrogen peroxide (H_2_O_2_), and hydroxyl radicals (OH^−^) [36]. The plant hormone ABA plays a critical role in abiotic stress and activates multiple stress-responsive genes [37]. Enhanced ABA levels have also shown to be correlated with drought stress in plants [38]. ABA is synthesized in plants from a carotenoid precursor (C_40_ carotene) and xanthoxin [39]. ABA levels considerably fluctuate during dehydration and rehydration. However, under such instances, the balance of ABA levels will be maintained by the catabolism of ABA. The high level of ABA accumulation can be catabolized by oxidation or conjugation reactions [40]. Under drought stress, the ABA is converted to 8’-hydroxy ABA and then isomerized to phaseic acid (PA). This process is catalyzed by ABA 8’-hydroxylase (ABA8Ox), an enzyme that belongs to the CYP707 family [41]. The physiological processes controlled by ABA in plants are achieved by the synergistic relationship between the biosynthesis and catabolism of ABA, which is mediated by ABA 8’-hydroxylases. In maize, the *CYP707A* (*ABA8Ox*) gene was found to upregulate when exposed to water deficit conditions [42]. Similarly, *CYP707A1* and *CYP707A2* genes were found to be significantly upregulated under osmotic stress in *Arachis hypogaea* [43] and *Populus simonii* (a highly drought-tolerant tree species found in China) [44]. The same genes (*CYP707A1* and *CYP707A2*) have also been reported to be upregulated under drought stress in *Arabidopsis* [40].

The CYPs have also been found to play a role in the synthesis of leaf lignin and grain formation when exposed to drought stress in plants. For example, CYP96A8 was speculated to be involved in lignin biosynthesis and other drought response-related functions [45]. Further, CYP86A2 plays a major role in the biosynthesis of epicuticular lipids such as cutin. Mutants of the *CYP86A2* gene in *Arabidopsis* exhibit a reduced cuticle membrane thickness and increased water permeability to help in drought tolerance [46]. The *LEAF CURLING RESPONSIVENESS* (*LCR)* gene, which encodes CYP86A8 in *Arabidopsis*, was found to be involved in the omega-hydroxylation of fatty acids in the biosynthesis of cutin [47]. Likewise, CYPs have also been reported to be involved in the biosynthesis of cuticular wax along with the *WXP1* gene in transgenic plants of *Medicago sativa* [48]. In response to drought stress, CYPs can directly or indirectly involve in the biosynthesis of several antioxidants, which can reduce the oxidative damage. A citrus CYP gene, *CsCYT75B1* was found to be upregulated during drought stress in *Citrus sinensis*; further, the transformation and overexpression of this gene in *Arabidopsis* significantly enhanced the total flavonoid content and antioxidant activity under drought stress [4]. The *CYP75* family was also found to be involved in flavonoid regulation in grapevine and ferns [49]. The heterologous expression of the *Carthamus tinctorius CYP82G24* gene in *Arabidopsis* induces the expression of several other genes involved in flavonoid biosynthesis [50]. Transcriptome analyses of sorghum plants found upregulation of the *CYP71A25* and *CYP71B2* genes under drought stress along with other drought-responsive genes [51]. Further, two CYPs have been identified to be upregulated in the rice variety Nagina 22 under drought stress, but the specific function of these CYPs have not been characterized [52]. Five uncharacterized CYP genes were found differentially expressed between drought-tolerant and -susceptible genotypes of rice when exposed to long-term drought stress [53].

### 3.2. Temperature Stress

Temperature fluctuations during plant growth and development are common. If plants are exposed to temperature variations, i.e., 10–15 °C above optimum (heat stress), <20 °C (chilling), or below 0 °C (freezing), for a prolonged period, it can result in irreversible damage to plant growth and development [54,55,56,57]. Both heat and cold stress generally affect respiration and photosynthesis, leading to oxidative damage caused by the production of ROS. The role of CYPs in the regulation of non-enzymatic antioxidants such as carotenoids, flavonoids, and hormones (e.g., abscisic acid) and the activation of antioxidant enzymes have been investigated. The expression of CYP genes involved in flavonoid production was found to be differentially regulated under heat and/or cold stress in *Lolium perenne* and *Festuca arundinacea*. The *CYP73A* (trans-cinnamate 4-monooxygenase), *CYP75A* (flavonoid 3’,5’-hydroxylase), and *CYP75B* (flavonoid 3’-monooxygenase) genes were significantly upregulated under heat and cold stress in both the species [58]. Prolonged cold stress in *Arabidopsis* induced a 2–4-fold expression of the CYP*83A1* gene involved in flavonoid (phenylpropanoids) metabolism [59]. ABA also can play a critical role in cold and heat stress response by increasing the expression of ABA 8’-hydroxylases (*CYP707A* genes) in *Arabidopsis* [60,61]. A *Panicum virgatum* population exposed to long-term heat stress (38/30 °C, day/night, for 50 days) showed a differential expression of 11 CYPs compared with those grown under ambient temperature. Specifically, two *CYP71A1* genes responsible for the biosynthesis of indole alkaloid secologanin were upregulated under heat stress [62]. In contrast, *CYP71* was highly downregulated in the leaves of *Rhazya stricta* exposed to a high-temperature range (40–42.4 °C) [63]. Secologanin is a monoterpene glycoside involved in the biosynthesis of alkaloids. A genome-wide association study of heat-tolerant *Brassica napus* identified that the *CYP71A23* gene is involved in pollen sterility [64]. The same gene was also upregulated in *Panicum maximum* exposed to elevated heat and CO_2_ [65]. A transcriptome analysis of a cold-tolerant sorghum genotype revealed the upregulation of two CYPs, the *CYP99A1* and *CYP709C1* genes [66].

### 3.3. Salinity Stress

Soils with a pH above 8.5 are considered saline, which can affect the crop yields significantly. The undesirable effects of salinity stress in plants include toxicity induced by the accumulation of sugars, amino acids, and various inorganic compounds, and osmotic stress by the reduced uptake of water [67]. Two major mechanisms involved in salinity tolerance in crops include leaf Na^+^ exclusion mediated by high-affinity K^+^ transporters (HKTs) and ROS homeostasis [68]. The manipulation of a CYP expression can impart tolerance to salt stress. Constitutively expressing the *TaCYP81D5* gene enhances the salinity tolerance in wheat both at the seedling and reproductive stages via accelerating the ROS scavenging activity [69]. The activation of the *AtCYP81D8* gene has been used as a marker for ROS activity [70]. CYPs also play a major role in maintaining ROS homeostasis to provide salinity tolerance. The induction of ABA is highly correlated with the salinity stress response. Low ABA levels are more adaptive for salinity stress [37]. CYP707A was found to be involved in ABA biosynthesis, and thus can indirectly protect plants from salt stress. Salicylic acid signaling is also found to enhance ABA synthesis in plants during salinity stress [71]. The *AtCYP709B3* gene was found highly expressed under salt stress in *Arabidopsis* providing tolerance to salinity [72]. An increased expression of the *CYP709* family was also found in salt-stressed *Robinia pseudoacacia* [73]. The proteome of NaCl-exposed *Physcomitrella patens*, naturally tolerant to high salinity, revealed the expression of 49 CYPs. Under salinity stress, the CYPs were speculated to reduce damage to the cell wall and scavenge the ROS [74].

### 3.4. Heavy Metal Stress

Metals such as aluminum (Al), iron (Fe), cobalt (Co), zinc (Zn), silver (Ag), cadmium (Cd), nickel (Ni), and mercury (Hg) with relatively high densities (>5 g/cm^3^) and toxic at a low concentration are referred to as heavy metals. The disposal of sewage sludge and other anthropogenic activities can increase the concentration of heavy metals in soil [75]. Not all the heavy metals are toxic to plants, and many of them—Zn, Fe, Co, etc.—at low concentrations are essential for plant growth and development. A transcriptome analysis of wheat varieties tolerant to aluminum toxicity resulted in the identification of an increased expression of the *CYP88A* gene (also known as *KAO1*), which is involved in gibberellin biosynthesis [76]. In a different study, *Pisum sativum* with the *KAO1* gene showed stunted growth [77]. The upregulation of multiple CYP genes has been reported in wheat genotypes susceptible to Al toxicity. *CYP81D8*, which is hypothesized to be involved in the metabolism of Al, showed a 4-fold expression in response to Al toxicity in *Arabidopsis* [78]. Two CYP genes (an uncharacterized CYP and *CYP99A1*) have been found to show a 32- and 21-fold increased expression, respectively, to Cd toxicity in rice. In some plants, CYPs are also believed to be involved in the metabolism of heavy metals along with other genes such as glutathione-*S*-transferase (GST) [79]. *Medicago sativa* plants transformed with the human *CYP2E1* and *GST* genes showed potential for phytoremediation in Hg-contaminated soils. Transgenic *M. sativa* expressing both these genes also had a synergistic effect that helped plants to withstand mercury contamination via enhanced metabolism [80]. In roots of *Panax ginseng* treated with Ni and Cd, the upregulation of the gene *CYP71*, involved in the biosynthesis of flavonoids, alkaloids, and other secondary metabolites has been reported [81].

### 3.5. Herbicide Stress

Plants metabolize xenobiotics such as pesticides that enter into their system; however, the ability to metabolize xenobiotics differs between and within different species. Herbicides are used extensively to control weeds in both crop and non-crop areas. The ability of crop plants to withstand herbicide applications targeted to control weeds is referred to as herbicide selectivity. CYPs are one of the key enzymes involved in conferring selectivity in crop plants via the metabolism of herbicides [82]. A novel mapping method, bulk segregant analysis combined with RNA-Seq (BSR-Seq), was used to map the gene *CYP81A9*, responsible for the metabolism of the acetolactate synthase (ALS) inhibitor herbicide nicosulfuron in maize [83]. Several rice varieties cultivated in Asia are naturally tolerant to the ALS inhibitor herbicide bentazon, and the genetic transformation of the rice *CYP81A6* gene to *Arabidopsis* and *Nicotiana tabacum* proved that CYP81A6 is involved in the metabolism of betazon [84]. The metabolism of herbicides such as chlorsulfuron, triasulfuron, metsulfuron-methyl, bensulfuron-methyl, and tribenuron-methyl by CYP71C6v1 from wheat was demonstrated by a heterologous expression in yeast [85]. Maize is naturally tolerant to 4-hydroxyphenylpyruvate dioxygenase (HPPD) inhibitor herbicides [86]. A CYP-mediated metabolism of the HPPD inhibitors mesotrione [87] and tembotrione [88] was reported in maize. Several uncharacterized CYP genes have been found to confer resistance to herbicides with different modes of action such as nicosulfuron (ALS inhibitor), mesotrione (HPPD inhibitor), dicamba (synthetic auxin), diflufenzopyr (auxin transport inhibitor), and carfentrazone (protoporphyrinogen oxidase (PPO) inhibitor) in maize [89,90]. CYPs are also involved in the metabolism of several herbicides in weeds, making them resistant to herbicides. The role of CYPs in the metabolism of herbicides in weeds has been reviewed extensively [82,91,92] and beyond the scope of this review.

### 3.6. Biotic Stress: Diseases, Insects, and Weeds

The metabolism of polyunsaturated fatty acids synthesizes oxylipins. The two major oxylipins in plants are jasmonic acid and methyl jasmonate. The expressions of genes responsible for the biosynthesis of these oxylipins and levels of these molecules in plants play an essential role in multiple stress signaling pathways, especially during physical injury and disease defense [93,94]. In plants, lipoxygenase is metabolized by several enzymes such as allene oxide synthase (AOS) and hydroperoxide lyase (HPL), which are members of the *CYP74* family [95,96]. Apart from the biosynthesis of oxylipins, CYPs are involved in the jasmonic acid and methyl jasmonate signaling pathways, for instance, in soybean, the *CYP82A3* gene expression is induced by methyl jasmonate which was also found to be induced by several fungal infections. Transgenic *Nicotiana benthamiana* plants overexpressing the *GmCYP82A3* gene were found to be highly resistant to black shank (*Phytophthora parasitica*) and gray mold (*Botrytis cinereal*) [3]. Hypersensitive response (HR) is a common defense mechanism for microbial infection in various crop species. The CYP gene *CaCYP1* from *Capsicum annuum* was found to be involved in the HR, following the infection by *Xanthomonas axonopodis* [97]. The CYP gene *AtCYP76C2* from *Arabidopsis* was found to be associated with hypersensitive rapid cell death, which is a defense mechanism for bacterial canker (*Pseudomonas syringae*) infection [98]. In the head blight-resistant wheat genotype “Ning 7840,” *CYP709C3v2* was upregulated along with the chitinase (*Chi1*) gene, which confers tolerance to the fusarium head blight (*Fusarium graminearum*) [99]. A pathogen-induced *CYP82C2* gene and other possible CYPs are involved in the biosynthesis of 4-hydroxyindole-3-carbonyl nitrile with cyanogenic functionality against bacterial canker (*Pseudomonas syringae*) [100]. Camalexin is a secondary plant metabolite which is involved in fungal and bacterial tolerance, where CYP71B15 catalyzes the reaction of dihydrocamalexic acid to form camalexin [101]. The CYP enzyme CYP96A15 (referred to as mid-chain alkane hydroxylase (MAH1)) is involved in epicuticular wax biosynthesis, which is a common structural plant defense mechanism. The upregulation of CYP transcripts during the wounding process has been recorded in *Helianthus tuberosus* [102], *Pisum sativum* [103], and maize [104].

Resistance to green peach aphid (*Myzus persicae*) in *Arabidopsis* was found to be controlled by the CYP family *PAD3* gene, which is involved in camalexin, known as a toxic phytoalexin [105]. The CYPs of a well-characterized *CYP79D* gene family were found to be involved in the herbivore-induced biosynthesis of aldomixes in *Populus trichocarpa* [106]. Cembratriene-ol (CBT-ol) in the trichome glands of *Nicotiana tabacum* plants was found to be converted into cembratriene-diol (CBT-diol) by an uncharacterized CYP hydroxylase, suppressing this CYP increased the CBT-ol content and exhibited resistance to aphids (*Myzus nicotianae*) [107]. CYPs’ involvement in the biosynthesis of cutin, lignin, and cyanogenic glucosides can directly or indirectly be associated with plant defense mechanisms against various biological threats [9].

Crop production is challenged by weed infestation resulting in an enormous crop yield loss due to crop–weed competition. The weeds compete with the crops for nutrients, water, and light. Weed control measures are focused directly or indirectly towards improving the competitive ability of the crop plants [108]. Allelopathy is defined as the effects (stimulatory or inhibitory) of a plant on the development of neighboring plants through the release of secondary compounds. Some crops express their allelopathic potential by releasing allelochemicals that suppress the weeds. Allelopathic effects have been documented for crops such as rice, wheat, sorghum, sunflower, rapeseed, and rye [109]. Sorgoleone (2-hydroxy-5-methoxy-3-[(Z, Z)-8’,11’,14’-pentadecatriene]-pbenzoquinone which is produced in root hairs of sorghum [110] can suppress some of the major problematic weeds of the United Stated such as *Amaranthus palmeri* [111], *Abutilon theophrasti* [112], *Echinochloa crus-galli* [113], and *Lolium rigidum* [114]. The CYP enzyme CYP71AM1 is involved in the biosynthetic pathway of the allelochemical sorgoleone in sorghum. CYP71AM1 catalyzes the formation of dihydrosorgoleone using 5-pentadecatrienyl resorcinol-3-methyl as a substrate in sorgoleone pathway [115]. Benzoxazinoids are specialized metabolites that are predominantly present in monocot species. Naturally occurring benzoxazinoids DIBOA [4-dihydroxy-2H-1,4-benzoxazin-3(4H)-one] and DIMBOA [4-dihydroxy-7-methoxy-2H-1,4-benzoxazin-3(4H)-one] are known to play a role in allelopathic plant–plant interactions and also serve as defense compounds against microbes, insects, and weeds [116]. DIBOA and DIMBOA have proven to inhibit the growth and development of weeds in crops such as wheat [117], maize [118], and rye [119]. Four CYPs belonging to the *CYP71* family play a key role in the biosynthesis of these compounds in wheat [120] and maize [121].

## 4. Conclusions

CYPs are versatile enzymes involved in multiple processes of plant growth and development and also play an essential role in stress response. CYPs protect plants from abiotic and biotic stresses by the biosynthesis and regulation of hormones, fatty acids, sterols, cell wall components, biopolymers, and several other defense compounds (terpenoids, alkaloids, flavonoids, furanocoumarins, glucosinolates, allelochemicals) [122]. Even though the involvement of several CYPs in different plant stress responses has been identified, the precise function of most of the CYPs is still elusive. The CYP genes involved in desirable plant functions can potentially be used to improve crop varieties, especially for stress tolerance [123]. A complete understanding of the biochemical processes catalyzed by a CYP along with the availability of sequence information are valuable for crop improvement by deploying marker-assisted selection, genetic transformation, or gene-editing techniques. In recent years, crop improvement programs have been focused on developing climate-smart crop varieties that can withstand abiotic stresses, although not by engineering the CYP genes that are known to provide resistance to abiotic stresses. The development of crop varieties by integrating abiotic and biotic stress resistance traits can significantly improve crop productivity [124]. Recently, several CYPs have been characterized and have the potential to be exploited in crop improvement to develop stress-tolerant crops (Table 1).

## Figures and Tables

**Table 1:** List of cytochrome P450 genes that can be used as candidates in crop improvement; all the list items given here are characterized for their biochemical process and involvement in plant function and traits desirable for crop improvement.

CYP	Identified Species	Biochemical Process	Function	Desirable Trait	Reference
*CYP97C1*	*Arabidopsis*	Carotenoid *ε*-ring hydroxylation	Lutein biosynthesis	Abiotic stress resistance	[125]
CYP703A2	*Arabidopsis*	Hydroxylation of lauric acid	Pollen development	Abiotic stress resistance	[126]
CYP83A1 and CYP83B1	*Arabidopsis*	Biosynthesis of glucosinolates	Pungency	Insect resistance	[127]
CYP79A1 and CYP71E1	Sorghum	Tyrosine into *p*-Hydroxymandelonitril	Cyanogenic glucoside (dhurrin) biosynthesis	Insect resistance	[128]
CYP72A1	*Catharanthus roseus*	Secologanin synthase	Indole alkaloid biosynthesis	Disease resistance	[129]
CYP707A	*Arabidopsis*	ABA 8’-hydroxylases	ABA regulation	Abiotic stress resistance	[38]
CYP86A2, A8	*Arabidopsis*	Omega-hydroxylation	Cutin biosynthesis	Insect resistance	[46]
*CYP714A3*	Rice	Gibberellin regulation	Shoot development	Heavy metal stress	[76]
CYP88A	Wheat	Gibberellin biosynthesis			
CYP86A1	*Arabidopsis*	Omega-hydroxylase	Suberin biosynthesis	Insect resistance	[130]
CYP71C	Maize	DIBOA biosynthesis	Allelopathy	Biotic stress resistance	[118]
